# Pathological Characteristics of Primary Bladder Carcinoma Treated at a Tertiary Care Hospital and Changing Demographics of Bladder Cancer in Sri Lanka

**DOI:** 10.1155/2016/5751647

**Published:** 2016-01-14

**Authors:** S. Sasikumar, K. S. N. Wijayarathna, K. A. M. S. Karunaratne, U. Gobi, A. Pathmeswaran, Anuruddha M. Abeygunasekera

**Affiliations:** ^1^Urology Unit, Colombo South Teaching Hospital, 10350 Dehiwala, Sri Lanka; ^2^Department of Pathology, Colombo South Teaching Hospital, 10350 Dehiwala, Sri Lanka; ^3^Department of Public Health, Faculty of Medicine, University of Kelaniya, 11010 Ragama, Sri Lanka

## Abstract

*Objectives*. The aim was to compare demographics and pathological features of bladder carcinoma treated in a urology unit with findings of previous studies done in Sri Lanka.* Materials and Methods*. Data of newly diagnosed patients with bladder cancer in a tertiary referral centre from 2011 to 2014 were analysed. Data on bladder cancers diagnosed from 1993 to 2014 were obtained from previous publications and Sri Lanka Cancer Registry.* Results*. There were 148 patients and mean age was 65 years. Male to female ratio was 4.1 : 1. Urothelial carcinoma (UC) was found in 89.2% of patients. Muscle invasion was noted in 35% of patients compared to 48.4% two decades ago. In patients with UC, 16.5% were found to have pT_1_ high grade tumour. It was 5.3% from 1993 to 2000. Pure squamous cell carcinoma was found in 8.1% of patients while primary or de novo carcinoma in situ (not associated with high grade pT_1_ tumours) was seen in one patient only.* Conclusions*. The percentage of squamous carcinoma is higher among Sri Lankan patients while primary carcinoma in situ is a rarity. The percentage of muscle invasive disease has decreased while the percentage of pT_1_ high grade tumours has increased during the last two decades in Sri Lanka.

## 1. Introduction

Sri Lanka is an island nation in South Asia, the others being India, Pakistan, Bangladesh, Nepal, Bhutan, and Maldives. Twenty-three urological surgeons serve the country's population of 20 million. Sri Lanka has been categorized as a middle income country recently and has a National Health Service which is free at the point of delivery. Though National Health Service provides healthcare to the vast majority of people, there are private sector hospitals which provide services to the more affluent. Average life expectancy at birth is 75 years in Sri Lanka and the country spends 3.3% of its gross domestic product for health [1]. Prostate and bladder cancers contribute to most of the urological cancers in Sri Lanka [2].

Bladder cancer is the seventh most common malignancy worldwide and the fourth common cancer in men [3]. In South Asia, reported rate of bladder cancer is about 2.1 per 100 000 [3]. In Sri Lanka it is 0.8 per 100 000 according to the Cancer Registry data compiled by the National Cancer Control Programme of Sri Lanka (NCCPSL) which is based on patients registered at oncology units of the country [2]. Hence incidence of bladder cancer appears to be low in Sri Lanka compared to rates in other South Asian countries such as India (3.2 per 100 000) and Karachi, Pakistan (8.9 per 100 000) [4, 5]. In Delhi, India, the age adjusted bladder cancer incidence reaches 5.8/100 000 [6].

Bladder cancer shows a male predominance with a sex ratio of 3 : 1 [3]. The spectrum of bladder cancer is diverse, but the majority (nearly 90%) are urothelial tumours. The other tumours are squamous cell carcinoma, adenocarcinoma, and rare varieties like small cell carcinoma. Clinical stage and grade are the two most important determinants of the prognosis of bladder cancer [7]. Previous studies show varying results in relation to sex ratio, proportion of muscle invasive disease, and histological types of bladder cancer in Sri Lanka and in neighbouring South Asian countries [2, 8–18]. Therefore the aims of our study were to identify the clinical and pathological characteristics of bladder cancer treated at a single urology unit in a tertiary hospital of Sri Lanka and to identify changes in demographics and pathological features of bladder carcinoma during the last two decades in Sri Lanka by perusing already published data. Since Sri Lankan Cancer Registry data is based only on patients registered at oncology units of the country, we aimed to compare its data with the data obtained from urology units of Sri Lanka to determine the accuracy and reliability of the available data regarding bladder cancer in Sri Lanka.

## 2. Materials and Methods

This descriptive study was performed on newly diagnosed primary bladder cancer patients at a urology unit of a tertiary care hospital (Colombo South Teaching Hospital, Sri Lanka) between 1 January 2011 and 31 December 2014. The data were collected from the bladder cancer register maintained in the unit. All consecutive patients who underwent their first transurethral resection of bladder tumour in the unit were included in the study. Pathological grading was assessed according to the World Health Organisation (WHO) and International Society of Urological Pathology (ISUP) classification 2004 [19]. Every attempt was taken to perform a complete resection of the tumour during the first surgery. Once the histology was available, those who did not have muscle tissue in the specimen were scheduled to have re-resection in six weeks' time. Thereafter patients were referred to the oncologist for appropriate further treatment. A contrast enhanced CT scan of kidneys, ureter, and bladder (KUB) was done in patients with muscle invasive disease.

All publications on bladder cancer in Sri Lanka after 1993 were identified by searching PubMed database as well as by perusing Sri Lankan journals manually which are not indexed in PubMed. Data on clinical and histopathological features of bladder cancers diagnosed from 1993 to 2014 were obtained from those publications and Sri Lanka Cancer Registry [2, 8–13, 20]. There were only three urology units in 1993 and by the end of 2014 there were 16 urology units treating bladder cancers in Sri Lanka. Statistical analysis was done using the Chi-square test. A* p* < 0.05 was considered statistically significant. Approval for the study was obtained from the Ethics Committee of the Institute.

## 3. Results

**Figure 1:**
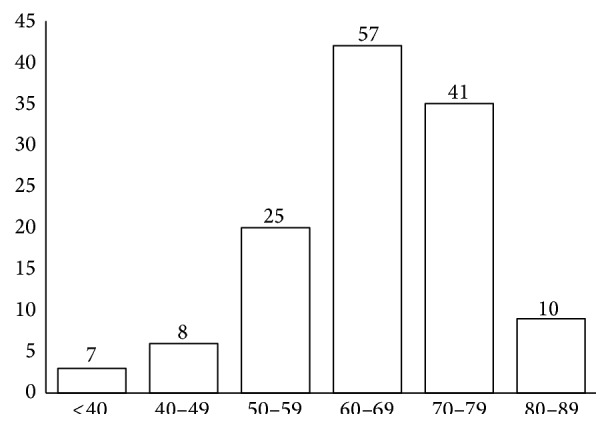
Age distribution of patients with bladder cancer.

There were 148 patients with newly diagnosed primary bladder malignancies during the study period of four years. One urachal carcinoma and one inflammatory myofibroblastic tumour of the bladder treated during the study period were not included in the study. Two more patients had metastatic deposits in the bladder from a breast carcinoma and a melanoma. They were also excluded from the study. Average age of the study cohort was 65 years (range 28–88) and 91% were more than 50 years old (Figure 1). The mean age was similar to that of other studies done in Sri Lanka from 1993 to 2011 (Table 1).

There were 119 men with a male to female ratio of 4.1 : 1. The male to female ratio was 5.5 : 1 and 9 : 1 in two studies done at National Hospital of Sri Lanka (NHSL) [11, 12]. Haematuria was the most common clinical presentation encountered in 81% (120/148) of patients. Others presented with lower abdominal pain (7%), lower urinary tract symptoms (7%) and found incidentally (5%). The initial investigation which indicated the diagnosis was urinary tract ultrasonography in 115 (77.7%) patients.

There were 132 (89.2%) urothelial tumours (Table 2). The proportion of urothelial tumours in the previous studies done in urology units of Sri Lanka ranges from 90.9% to 97% (Table 2). However according to the Cancer Registry maintained by NCCPSL, only 72% and 69% were urothelial tumours during the period from 2001 to 2005 and in 2006, respectively [10, 13]. This difference was statistically significant (*p* < 0.05).

Staging could not be completed in 5 patients with urothelial carcinoma as they could not come for re-resection at six weeks when there was inadequate muscle in the initial specimen. Therefore 127 patients with urothelial carcinoma were included in the analysis for staging. Majority (54.3%) of the urothelial tumours were in the T_1_ stage (Table 3). About 35% of the staged patients had muscle invasive disease. According to the study done by Goonewardena et al. at NHSL from 1993 to 2000, 48.4% were muscle invasive tumours at initial presentation (Table 1) [11]. According to the data from the Cancer Registry of the NCCPSL, 94% had muscle invasive disease (Table 3) [10].

There were 21 high grade malignancies among the 69 T_1_ stage urothelial tumours (Table 3). Therefore in our study, the percentage of pT_1_ high grade tumour among urothelial cancers was 16.5% and is closer to the findings of a study done at NHSL in 2010 which was 18% [9]. A study done in the same urology unit of the NHSL from 1993 to 2000 showed the pT_1_ high grade tumour to be only 5.3% and this difference was statistically significant (*p* < 0.005) [11]. Among muscle invasive urothelial cancers, 86.4% (38/44) were high grade tumours.

Urothelial carcinoma with squamous differentiation was found in 11 (7.4%) of patients and all those patients were found to have muscle invasive and high grade tumour in our study. There were one urothelial tumour with glandular differentiation and one micropapillary variety. Among noninvasive urothelial carcinomas there was one primary (de novo) urothelial carcinoma in situ and one noninvasive papillary urothelial neoplasm of low malignant potential in our study (Table 3). Cancer Registry data from 2001 to 2005 shows only 10 cases (1.9%) of carcinoma in situ of the bladder [20]. None of the other published studies done in Sri Lanka show any cases of primary or de novo urothelial carcinoma in situ of the bladder (Table 1). Therefore primary carcinoma in situ not associated with pT_1_ high grade tumours is extremely rare in Sri Lanka.

Squamous cell carcinoma was present in 8.1% (12/148) of patients. None of the patients with squamous cell carcinoma had bladder stones at the time of diagnosis while only one had surgery for bladder stones done 20 years ago. Furthermore they had not travelled outside Sri Lanka. Although the percentage of urothelial tumours in the present study was similar to published data from other urology units, the percentage of squamous cell carcinoma in the present study (8.1%) was significantly higher (*p* = 0.009) than the percentage (3%) reported from NHSL (Table 1) [11, 12]. According to the Cancer Registry, percentage of squamous cell carcinoma in Sri Lanka was 9% which was closer to the percentage of the present study [2, 13].

In the present study there were two patients (1.4%) with adenocarcinoma of bladder, which was in par with the 1.3% of the study done at NHSL [11]. Both were of enteric type. There was one lymphoma and immunohistochemistry showed it to be a large B cell lymphoma. A 34-year-old female patient had a leiomyosarcoma of the bladder.

## 4. Discussion

The average age at diagnosis of bladder cancer patients in Sri Lanka is similar to that of India which is around 65 [15]. In Pakistan the mean age of bladder cancer is around 55–58 years [17, 18]. In the present study male to female ratio was 4.1 : 1, compared to the ratio of 3 : 1 quoted worldwide [3, 21]. In India the male to female ratio is 8.6 : 1 [16]. All Sri Lankan studies show a higher male preponderance similar to other South Asian countries than described in the western countries [16–18]. Low prevalence of smoking among women in Sri Lanka could be the reason for this difference [1].

The proportion of urothelial tumours among bladder cancers in our study cohort (89.2%) is less than those of India and Pakistan which are over 95% [15–18]. The reason for this is the higher percentage of squamous cell carcinoma seen in Sri Lanka. In the western world the percentage of squamous cell carcinoma among bladder cancers is around 1–3%, depending on the ethnicity of the population [16, 22]. The present study involving 148 patients and data from National Cancer Registry show a significantly higher percentage (around 8%) of squamous cell carcinoma. Even in neighbouring India percentage of squamous cell carcinoma among bladder cancers is low [16]. While the percentage of urothelial carcinoma and adenocarcinoma remain the same among studies, the reason for the significantly high percentage of squamous cell carcinoma of the bladder among patients treated outside NHSL is unclear. Since schistosomiasis is not found in Sri Lanka, the reason for high percentage of squamous cell carcinoma of bladder is only speculative. One plausible explanation could be the agrochemicals that are being used commonly in Sri Lanka by its semiurban and rural populations. Sri Lanka has one of the highest rates of pesticide use in the world and is believed to be a reason for the high incidence of chronic kidney disease of uncertain origin in Sri Lanka [23, 24].

Stage pT_1_ high grade urothelial tumour also known as high grade lamina-invasive bladder cancer is unique in its aggressive behaviour [25]. It shows a high recurrence rate and commonly progresses to invasive disease [26]. The percentage of pT_1_ high grade tumours has increased over the last two decades in Sri Lanka (Table 1). One possible reason for this difference could be the change in the pathological grading system over the years from 1973 WHO classification to 2004 WHO classification. The 1973 WHO classification which was used in the earlier study had three different grades of anaplasia (G1, G2, and G3 or high grade) while the 2004 WHO classification which was used in the present study has only two grades of anaplasia (low and high grades).

According to a study done at NHSL from 1993 to 2000, the percentage of muscle invasive tumours at initial presentation was 48.4% but the corresponding value was 35% in the present study (Table 1) [11]. The percentage of muscle invasive disease in our study (35%) is similar to that of Manipur in India (36.36%) and Pakistan (37.6%) [14, 18]. This gradual decline in the percentage of muscle invasive tumours could be due to the early diagnosis of the disease due to improving healthcare facilities and urological services in Sri Lanka. It is known that the depth of tumour invasion in the bladder wall could be time dependent [27].

Urothelial tumours with squamous or adenodifferentiation are more aggressive and are at a higher stage at initial presentation [28]. In the present study there were eleven patients with squamous differentiation and one patient with glandular differentiation and all patients had muscle invasive, high grade tumours at presentation. Percentage of adenocarcinoma among patients with bladder cancer in Sri Lanka is in par with the percentage seen in the western world which is around 2% [29]. However the occurrence of primary carcinoma in situ not associated with pT_1_ high grade tumours in the bladder appears to be extremely rare in Sri Lanka similar to India [14]. The reason for the very low incidence of primary carcinoma in situ in South Asian countries is unknown. A protective factor in the South Asian diet rich in spices or a genetic variation is a possible reason.

Many patients with noninvasive bladder cancer do not seek services of oncology units of Sri Lanka. They prefer to stay away from oncology units due to sociocultural reasons and stigma attached to cancer patients in South Asian countries like Sri Lanka. Hence their data are not included in the Cancer Registry of the NCCPSL. This could be the reason for the unusually high rate of muscle invasive disease and the lower percentage of urothelial tumours in the Cancer Registry of the NCCPSL when compared to data from urology units of the country.

Maintaining a comprehensive bladder Cancer Registry is essential to analyse the changing patterns of prevalence, incidence, survival, and mortality of malignancies. The different findings among the institutional studies need to be compared with nationwide trends. As the National Cancer Registry is based on data collected from oncology units and reporting of cancers by clinicians is not mandatory in the country, the data seems to be incomplete as evidenced by our analysis which shows significant differences in the patterns of the Cancer Registry and those of urology units. In Sri Lanka initial surgical management (transurethral resection of bladder tumour) of bladder tumours is always done in urology units and data from urology units are more likely to be representative in relation to clinicopathological characteristics. Therefore conclusions should not be made purely based on Cancer Registry data of Sri Lanka. Until a comprehensive electronic database is established, it is important for individual urology units in Sri Lanka to maintain their own cancer audits. It is time for all cancer patients in Sri Lanka to be referred to oncologists during treatment irrespective of the stage. When Sri Lankan healthcare planners use data, they must rely on a combination of data from Cancer Registry as well as individual units. Otherwise interpretations made could be erroneous and biased. Therefore with comparatively high smoking rates [1] and possible underreporting to oncology units, the incidence of bladder cancer is likely to be higher than the reported rate (0.8 per 100 000) in the National Cancer Registry of Sri Lanka.

In conclusion, the percentage of muscle invasive disease has decreased while the percentage of pT_1_ high grade tumours has increased during the last two decades in Sri Lanka. The incidence of squamous cell carcinoma is higher among Sri Lankan patients with bladder carcinoma while primary carcinoma in situ is a rarity. The reasons for some of these differences are uncertain and warrant further research in the identified areas.

## Figures and Tables

**Table 1:** Summary of results from published studies on bladder cancer in Sri Lanka.

Study (Time period of study and authors)	1993–2000 Goonewardena et al. [11]	2000-2001 Perera et al. [12]	2001–2005 Ranasinghe et al. [10] and NCCPSL [20]	2006 NCCPSL [13]	2007 NCCPSL [2]	2009 Sathesan et al. [8]	2010 Prabath et al. [9]	2011–2014 Present study
Male : female ratio	5.5 : 1	9 : 1	4 : 1	3 : 1	3.2 : 1	3.1 : 1	3.7 : 1	4.1 : 1
Average age	64	Not available	Median age group 62–64	65	Median age group 65–69	Median age 65	Median age 67.5	65
Urothelial carcinoma	93.4%	96%	72%	69.5%	79%	97%	90.9%	89.2%
Squamous carcinoma	3%	Not available	9%	9%	8%	0%	6.1%	8.1%
Adeno carcinoma	1.3%	Not available	3%	5%	4%	0%	0%	1.4%
Muscle invasive tumour	48.4%	30.9%	94%	Not available	Not available	37.3%	21.2%	35%
Primary carcinoma-in-situ	None	Not available	1.9%	None	None	None	None	0.7%
pT_1_ high grade tumour	5.3%	Not available	Not available	Not available	Not available	3%	18%	16.5%
Total number of patients	301	139	637	131	151	35	33	148

NCCPSL = Cancer Registry of National Cancer Control Programme of Sri Lanka.

**Table 2:** Histological classification of tumours included in the study cohort.

Tumour type	Number
Urothelial tumours (*n* = 132)	
Infiltrating urothelial carcinoma	105
With squamous differentiation	11
With glandular differentiation	1
Micropapillary	1
Noninvasive urothelial neoplasms (14)	
Urothelial carcinoma in situ	1
Noninvasive papillary urothelial carcinoma, low grade	12
Noninvasive papillary urothelial neoplasm of low malignant potential	1
Squamous neoplasms (*n* = 12)	
Squamous cell carcinoma	12
Glandular neoplasms (*n* = 2)	
Adenocarcinoma (enteric type)	2
Mesenchymal tumours (*n* = 1)	
Leiomyosarcoma	1
Haematopoietic and lymphoid tumours (*n* = 1)	
Lymphoma (large B cell)	1
Total	**148**

**Table 3:** Different pT stages of urothelial tumours.

Stage	Number
pT_a_ (*n* = 14)	
Low grade	12
High grade	2
pT_1_ (*n* = 69)	
Low grade	48
High grade	21
≥pT_2_ (*n* = 44)	
Low grade	6
High grade	38
Uncertain	5
Total	** 132**
